# Complete Genome Sequence of a New Maize-Associated Cytorhabdovirus

**DOI:** 10.1128/genomeA.00591-17

**Published:** 2017-08-03

**Authors:** Kristen Willie, Lucy R. Stewart

**Affiliations:** a USDA-ARS Corn, Soybean and Wheat Quality Research Unit, Wooster, Ohio, USA; b Department of Plant Pathology, Ohio State University, OARDC, Wooster, Ohio, USA

## Abstract

A new 11,877-nucleotide cytorhabdovirus sequence with 6 open reading frames has been identified in a maize sample. It shares 50 and 51% genome-wide nucleotide sequence identity with northern cereal mosaic cytorhabdovirus and barley yellow striate mosaic cytorhabdovirus, respectively.

## GENOME ANNOUNCEMENT

The family *Rhabdoviridae* includes viruses with plant, insect, fish, and mammalian hosts. Members of the genera *Cytorhabdovirus* and *Nucleorhabdovirus* are plant-infecting viruses that also infect their insect vectors (1). Here, we report the complete genome sequence of a new rhabdovirus we call “maize-associated cytorhabdovirus” discovered through RNA-Seq analysis of maize samples. The virus has a genome of 11,877 nucleotides (nt) with 6 open reading frames (ORFs). It is most similar to cytorhabdoviruses, sharing 51% genome-wide nucleotide sequence identity with the 12,707-nt barley yellow striate mosaic cytorhabdovirus (BYSMV; GenBank accession no. NC028244) genome, and 50% genome-wide nucleotide sequence identity with the 13,223-nt northern cereal mosaic cytorhabdovirus (NCMV; GenBank accession no. NC002251) genome.

Total RNA was isolated from lyophilized leaf tissue from two maize plants collected from a field in Lima, Peru, testing positive for infection with maize chlorotic mottle virus (MCMV) (2). Sample RNA was purified using a DirectZol RNA miniprep kit (Zymo Research, Irvine, CA, USA). RNA quantity and quality were determined using the Experion automated electrophoresis system (Bio-Rad, Hercules, CA, USA). After sequencing with the Illumina HiSeq platform (Illumina, San Diego, CA, USA), sequences with similarity to known plant viruses, including near-full-length sequences of MCMV and a unique sequence with highest similarity to grass-infecting cytorhabdoviruses, were identified using protocols outlined previously (2, 3). The cytorhabdovirus sequence was not identified in any other maize samples analyzed in the same study (2). Sanger-sequenced and amplified fragments from nt 3835 to 4308 and nt 4764 to 5591 were identical to the *de novo* assembled contig. The 5′ and 3′ end sequences were determined using First Choice RLM-RACE (Invitrogen-Thermo Fisher Scientific, Carlsbad, CA, USA; 5′ RACE primers: 5′ GCCCAAAACTGGAGACTAAAATGG 3′ and 5′ TTGAACCAGATGCTAAGAAGAGGG 3′; and 3′ RACE primers 5′ CACTGAAGAAGGTGGAGTAGCG 3′ and 5′ CTTCTGGCCCATATTCCTGA 3′).

Antisense ORFs 1 to 6 were predicted in the virus genome sequence at nt 7 to 1344, 1449 to 2345, 2462 to 2971, 3093 to 3596, 3663 to 5123, and 5405 to 11605. This is consistent with predicted ORFs of other described plant-infecting rhabdoviruses that contain 6 to 8 ORFs from the antisense RNA encoding the N, P, 3, 4 (variable), M, G, and L proteins. Predicted proteins were aligned to corresponding cytorhabdovirus proteins (MacVector, Apex, NC, USA) using ClustalW, with a Gonnet matrix for similarity calculations. The putative L protein is 60% identical and 76% similar to the L protein of BYSMV, 59% identical and 76% similar to NCMV L, and 70% identical and similar to the aligned portion of partial L protein of maize yellow striate virus (GenBank accession no. JQ715419). Translated ORFs 1 to 5 of the maize-associated rhabdovirus are 29.8 to 42.1% identical (50.2 to 60.2% similar) to corresponding proteins of BYSMV and 30.3 to 43.5% identical (48.4 to 62.1% similar) to NCMV proteins.

Previously described maize-infecting rhabdoviruses include maize mosaic virus (4), maize fine streak virus (5), and NCMV (6), which are transmitted by planthoppers or leafhoppers (7). The host range, symptoms, and transmission vector for the maize-associated cytorhabdovirus have not been identified yet.

### Accession number(s).

The genome sequence of maize-associated cytorhabdovirus has been deposited at GenBank under the accession number KY965147.
